# Non-Destructive Evaluation of the Leaf Nitrogen Concentration by In-Field Visible/Near-Infrared Spectroscopy in Pear Orchards

**DOI:** 10.3390/s17030538

**Published:** 2017-03-08

**Authors:** Jie Wang, Changwei Shen, Na Liu, Xin Jin, Xueshan Fan, Caixia Dong, Yangchun Xu

**Affiliations:** Jiangsu Provincial Key Lab for Organic Solid Waste Utilization; Ministry of Agriculture, Key Laboratory of Plant Nutrition and Fertilization in Low-Middle Reaches of the Yangtze River; College of Resources and Environmental Sciences, Nanjing Agricultural University, Nanjing 210095, China; 2014203044@njau.edu.cn (J.W.); 2014203026@njau.edu.cn (C.S.); 2014103109@njau.edu.cn (N.L.); 2014103108@njau.edu.cn (X.J.); 2014103110@njau.edu.cn (X.F.); ycxu@njau.edu.cn (Y.X.)

**Keywords:** non-destructive, visible/near-infrared spectroscopy, leaf nitrogen, PLSR, pear

## Abstract

Non-destructive and timely determination of leaf nitrogen (N) concentration is urgently needed for N management in pear orchards. A two-year field experiment was conducted in a commercial pear orchard with five N application rates: 0 (N0), 165 (N1), 330 (N2), 660 (N3), and 990 (N4) kg·N·ha^−1^. The mid-portion leaves on the year’s shoot were selected for the spectral measurement first and then N concentration determination in the laboratory at 50 and 80 days after full bloom (DAB). Three methods of in-field spectral measurement (25° bare fibre under solar conditions, black background attached to plant probe, and white background attached to plant probe) were compared. We also investigated the modelling performances of four chemometric techniques (principal components regression, PCR; partial least squares regression, PLSR; stepwise multiple linear regression, SMLR; and back propagation neural network, BPNN) and three vegetation indices (difference spectral index, normalized difference spectral index, and ratio spectral index). Due to the low correlation of reflectance obtained by the 25° field of view method, all of the modelling was performed on two spectral datasets—both acquired by a plant probe. Results showed that the best modelling and prediction accuracy were found in the model established by PLSR and spectra measured with a black background. The randomly-separated subsets of calibration (*n* = 1000) and validation (*n* = 420) of this model resulted in high R^2^ values of 0.86 and 0.85, respectively, as well as a low mean relative error (<6%). Furthermore, a higher coefficient of determination between the leaf N concentration and fruit yield was found at 50 DAB samplings in both 2015 (R^2^ = 0.77) and 2014 (R^2^ = 0.59). Thus, the leaf N concentration was suggested to be determined at 50 DAB by visible/near-infrared spectroscopy and the threshold should be 24–27 g/kg.

## 1. Introduction

Fertilization is considered one of the most effective ways to increase fruit yield and improve its quality. Among the 17 essential nutrients required by plants, nitrogen (N) is one of the key elements for pear tree growth, fruit yield, and quality. Generally, the N demand is relatively large compared with that of other elements. However, the positive response of tree vegetative and reproductive organs to added N triggered a higher application of N fertilizer without paying more attention to actual N requirement of trees, which might lead to an excessive N status in pear trees. The over-application of N not only results in vigorous vegetative growth and decreased sugar-to-acid ratio [1], but also increases the risk of nitrate leaching from soil, which contributes to the eutrophication of surface waters and degradation of drinking water quality. An excessive soil N availability in summer may delay fruit maturation, negatively impact the total soluble solids (major component is soluble carbohydrate) in fruit, and decrease plant tolerance to pests and diseases, such as psylla (*Cacopsylla pyri* L.) on “Bartlett” and post-harvest blue mould (*Penicillium expansum*) on “Conference” pears [2]. Synchronizing N availability with fruit N requirements offers the potential to protect the environment without sacrificing production [3].

Tissue testing is one tool that can aid in fertilizer management by evaluating N status and identifying fruit requirements for additional N. The measurement of traditional nutrient status by tissue testing was based on the destructive sampling of leaves, branches and fruits, sometimes even full tree excavation [4,5]. The determination of N concentration is labour-intensive and time-consuming in addition to being cost prohibitive (elemental analyser method) and requiring dangerous chemical reagents (Kjeldahl method) which may cause environmental contamination. Recently, with the rapid development and improvement of spectroscopy techniques, monitoring leaf reflectance or the transmittance of radiant energy can be considered a form of rapid, non-destructive tissue testing [6,7]. Visible near-infrared spectral reflectance data can be used to evaluate the leaf N concentration and chlorophyll content of cucumber [8], tomato [9], oilseed rape [10] and pear tree [11] using a specific regression method. However, these studies were generally performed on detached leaves under laboratory conditions without considering variable leaf N status, threshold and diagnosis time, and rarely considering about different models. Moreover, in-field estimating leaf nitrogen using hyperspectral data at canopy scale were mainly found on the trees or crops [12,13]. Hence, research is required on developing a similar technology for in-field, non-destructive leaf hyperspectral sensing of pear trees. 

Under steady laboratory conditions, spectral measurements are often conducted with integrating sphere because both reflectance and transmittance of leaves can be obtained at the same time. However, it’s too time-costing for the in-field measurement of leaf spectra. For an in-field spectral measurement, method of 25° bare optic fibre was usually used in the situation of whole plant level/the canopy measurement under the solar condition. If the leaf spectra were measured with a plant probe, the black background and the white background were found to be both used and reputable in many studies [14,15]. These in-field measurements mentioned above have their own advantages and disadvantages. For one thing, the solar condition is widely used for the in-field spectral measurement owing to the simple measuring device component but the light intensity is always changeful; while an internal and calibrated light of the plant probe is considered to be a steady light source but the measuring time is limited by the battery capacity during the field operation. For another, we are unable to remove the dust on the adaxial surface and the underside of leaves, so that the spectra obtained by using the leaf clip with black and white backgrounds should be compared to improve the signal-to-noise ratio of spectral data. Besides, suitable techniques should be used to make use of hyperspectral data. The techniques must deal efficiently with the strong multi-collinearity present in the spectral data and should not be too sensitive to sensor noise [16]. The methods such as principal components regression (PCR), partial least squares regression (PLSR), stepwise multiple linear regression (SMLR), and back propagation neural network (BPNN) are found to be good chemometrics methods to dealing with the spectral data [17,18]. In addition to these chemometric techniques, the remote sensing community developed over the past four decades a wide range of spectral indicators (e.g., vegetation indices, red-edge indices), responding strongly to the main vegetation biophysical variables, such as leaf area index and leaf pigmentation [19,20,21,22,23]. A number of spectrometric studies have been undertaken on plant N content monitoring using the vegetation indices and the red-edge [24,25,26]. However, the four chemometric techniques (PCR, SMLR, PLSR and BPNN) and vegetation indices for modelling the N concentration of pear leaves from spectrometer data collected in the field have not been tested so far.

A non-destructive estimate of yield was mainly conducted by collecting canopy spectra of a satellite platform and airborne hyperspectral scanners. Examples included the prediction of biomass and yield of winter wheat under different nitrogen supplies using spectral indices [27] and the grain yield prediction using reflectance spectra of canopy [28]. However, there is a lack of sensors on satellite platforms with optimal spatial resolution to monitor orchard crops at the tree scale [29]. Therefore, the estimation of fruit yield by the canopy spectra in the orchard often had a low R^2^, for example 0.33 in peach [29] and 0.58 in citrus [30]. 

In the present study, the best method to non-destructively evaluate the leaf nitrogen concentration by in-field visible/near infrared spectroscopy will be established. In addition, the right time for yield estimation by the leaf N status and the reference threshold of leaf N status for fertilization decisions will be find out. 

## 2. Materials and Methods

### 2.1. Plant Material and Treatments

A two-year field experiment was conducted from 2014 to 2015 in a commercial pear orchard in Gaochun (Jiangsu Province, China; 32°03′ N, 118°46′ E). The experimental site has an annual mean temperature of 15.9 °C and receives 1157 mm of precipitation. The soil has a clay-loamy texture with 69 mg·kg^−1^ alkali-hydrolysable nitrogen [31], 43 mg·kg^−1^ available phosphate by the method of Olsen [32], 146 mg·kg^−1^ available potassium by the method of Sommers [33], 17 g·kg^−1^ organic matter, and a pH 6.8 in water. Pear trees (*Pyrus communis* L.) that had been managed by trellis cultivation in a 3 m × 5 m frame for 12 years were used in the field experiment during the growing period (May to June). Referring to common N fertilization amounts in the region (660 kg·N·ha^−1^), six N fertilization treatments consisting of 0 (N0), 165 (N1), 330 (N2), 660 (N3), and 990 (N4) kg·N·ha^−1^ as urea (46% N) were separately split-applied four times (post-harvest, late autumn, pre-bloom stage and expansion stage) at 20%, 40%, 20%, and 20% of the total amount, respectively. Calcium superphosphate (12% P_2_O_5_) was applied at 528 kg·ha^−1^ only in late autumn as base fertilizer. In addition, for the quality of pear fruit, 990 kg·ha^−1^ of potassium sulphate (45% K_2_O) was applied three times (late autumn, pre-bloom stage, and expansion stage) at 40%, 20%, and 40% of the total amount, respectively. 

In a completely randomized block design, three replicates of three trees each were arranged in three alternate tree rows. The control comprised of five trees. From late autumn 2013 to the post-harvest stage of 2015, fertilizer was applied to six holes (30 cm wide × 30 cm deep) in a circle around each tree. The standard cultural practices used in local commercial production, including pruning, irrigation, and pest control were employed. Every season, the fruit crop load was manually adjusted by large-scale thinning to ensure high commercial quality.

### 2.2. Spectra Collection

**Figure 1:**
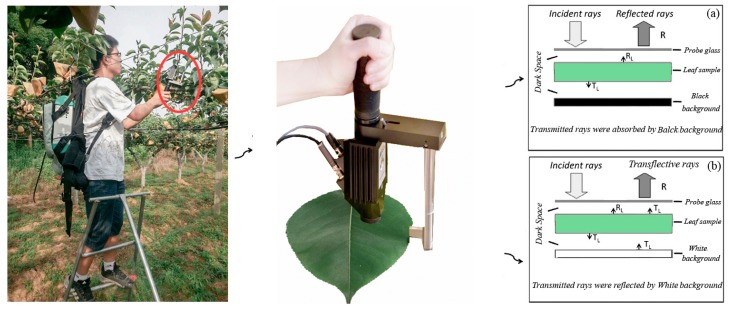
The schematic diagram of spectral acquisition by two methods. The reflectance measured with the black background (**a**); and the transflectance measured with the white background (**b**).

As reported by Tagliavini et al., the remobilization of nitrogen ceased between petal fall and the beginning of pear fruit development [34]. Nitrogen was transported into shoot leaves several weeks after bloom; shoot leaves are more dependent than spur leaves on spring N uptake. Therefore, the N concentration in new shoot leaves is sensitive to the N supply and can be used to evaluate the N status of the whole tree. The middle leaves of the year’s spring flush from the external side (east, south, west, and north) of the canopy were collected at 50 days (May) and 80 days after full bloom (80 DAB, June) for the spectral measurement. Eight or twelve leaves facing four directions from each tree were measured to represent the N status of the whole tree. In-field spectral measurements were achieved using a portable field spectrometer FieldSpec 3 (Analytical Spectral Devices, Boulder, CO, USA). The instrument covered wavelengths of 350–1000 nm (with a sampling interval of 1.4 nm and a spectral resolution of 3 nm) and 1000–2500 nm (with a sampling interval of 2 nm and a spectral resolution of 10 nm). The output spectral band number was 2151, and the interval of re-sampling was 1 nm. Before measuring each tree, Teflon white standard (Spectralon, Labsphere Inc., North Dutton, NH, USA) was used to set up the maximum reflectance (99.9%) conditions. The reflectance of each leaf was determined from the measurement of leaf radiance divided by the radiance of the reflective white standard. Three spectral measurement methods were compared: (1) 25° bare fibre under solar conditions, which was restricted by weather (sunny, cloudless days only, from 10:00 a.m. to 3:00 p.m.); (2) a leaf-clip assembly attached to a plant probe with an internal, calibrated light source (the transflectance of each leaf was determined using the white background of the leaf-clip); and (3) the black background of leaf-clip to determine absolute reflectance of each leaf (Figure 1). Two central symmetrical points on the leaf adaxial surface were designated as the measurement points on all leaves. The scan number at a given position was set to five, and the average value of 10 spectra was used as the final reflectance of the leaf.

### 2.3. Modelling Methods

Before modelling, preprocessing methods were used on the raw spectra. Wavelengths with very large atmospheric influence were removed for the 25° field of view measurements under solar conditions. Normalization was used on the raw spectra collected by the probe attached with backgrounds using Unscrambler X 10.3 (Camo Software AS, Oslo, Norway). Using the spectral data and N concentration, we investigated the predictive power of principal component regression (PCR), stepwise multiple linear regression (SMLR), partial least squares regression (PLSR), back propagation neural network (BPNN), and vegetation indices (difference vegetation index, ratio vegetation index, and normalized differential vegetation index). Full-spectrum methods as PCR, PLSR, and BPNN use all available wavelengths simultaneously [16]. In contrast, SMLR selects useful wavelengths from the available spectrum and ignores the remaining wavebands during model application.

The PCR and PLSR methods were analysed comparatively using Unscrambler X 10.3. The model obtained the optimal number of factors by leave-one-out cross-validation and the mean square error in calibration. 

The data compression stage of the SMLR consists of selecting a combination of a few spectral bands as regression factors. The regression factor matrix is then used to calculate the biophysical loadings by ordinary least square. In the present study, the wavelengths were first regressed sequentially against the biophysical variables. The wavelength with the highest explained variance was then chosen as the first regression factor. With this first regression factor fixed, the next wavelength was chosen, and so on. Forward and backward elimination were not investigated. SMLR was calculated by SPSS 20.0 (SPSS Inc., Chicago, IL, USA).

The back propagation neural network (BPNN), based on principal component analysis (PCA), was used to develop the models for predicting the leaf N concentration. For the PCA-BPNN models, PCA was performed first to extract information from the whole spectral regions, and some principal components which carried over 80% information of the original dataset were used as the neurons of the network input layer. Several network architectures were tested by varying the number of neurons in the hidden layer with different initial weights (at least 10 times). The optimal parameters of the target error, the training rate, and the iteration were determined by the least prediction error [35].

As a baseline method, linear regression models between N concentration and vegetation indices (DVI, RVI, and NDVI) were analysed. For the fast analysis and feature extraction from the observed datasets, we used the report by Yao [36]. The reduced sampling method was used to construct a difference spectral index (DSI), normalized difference spectral index (NDSI), and ratio spectral index (RSI). In this procedure, the spectral reflectance data were read at intervals of 10 nm within the range of 350–2500 nm. With the duo matrix formulation, all of the possible DSI, NDSI, and RSI based on any two individual bands at the interval of 10 nm from 350–2500 nm were regressed against an N concentration linear equation. According to the changing coefficients of determination (R^2^), a contour map of R^2^ was plotted. From this map, a sensitive spectral range with a relatively high R^2^ was identified. The vegetation index and BPNN were calculated in MATLAB R2012b (MathWorks, Natick, MA, USA).

The quality of the calibration model was evaluated using the following statistical parameters: coefficient of determination between predicted and measured N concentrations (R^2^), root mean square error in calibration (RMSEC), and root mean square error in validation (RMSEV). As mentioned by Saeys [37], a calibration model with an R^2^ value greater than 0.91 is considered to be excellent, whereas R^2^ between 0.82 and 0.90 represents good prediction. A small difference between the RMSEC and RMSEV values is also important to avoid ‘over-fitting’ in the calibration and validation phase [38]. 

### 2.4. Data Pre-Treatment 

No attempts were made to reduce the number of input variables through feature selection in order to compare the modelling accuracy of the seven methods. Before modelling, PCA was used to eliminate the outliers based on the spectral principal component analysis of 720 samples [11]. We found a large deviation in the spectra of most samples. After elimination, 710 samples were divided into the calibration set and the validation set using a randomly-selected collection for analysis (Table 1).

### 2.5. Leaf Nitrogen Concentration and Yield Estimation

The total N concentration of the leaf samples was measured analytically as a reference for the spectral prediction models. After spectral measurements were taken, the leaves were picked off the tree, sealed in valve bags, and taken to the laboratory for analysis. All of the leaves were first placed in a forced-air oven at 105 °C for 30 min and then dried to a constant weight at 70 °C for 48 h. The samples were finely ground (100 mesh) and analysed for the total nitrogen concentration using an Elementar Vario Macro CHN analyser (Elementar Analysensyteme GmbH, Hanau, Germany). A standard citrus leaf sample (GBW10020) was included to ensure accuracy of N concentration measurements. 

The fruit number per tree was counted at both the fruit expansion stage and the maturity stage to ensure the reliability of fruit number. Sixteen pear fruits were collected per tree at maturity and weighed to estimate the average single fruit weight per tree. The yield was calculated by multiplying the single fruit weight by the fruit number as kg per tree. N concentration of each treatment was obtained by an average of three replications’ leaf N concentration. Moreover, we performed polynomial curve fitting between the chemically-measured leaf N concentration and the yield to find the better time estimating the yield.

## 3. Results

### 3.1. Establishment of the Non-Destructive Measurement

**Figure 2:**
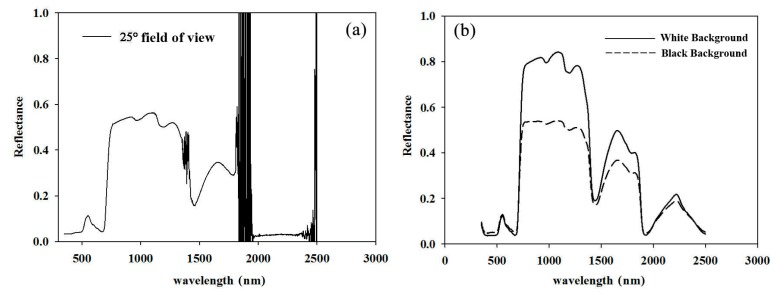
The characteristics of reflectance spectra of pear leaves by in-field spectral measurements using the 25° field of view (**a**); and either black background or white background (**b**) using the leaf clip.

The characteristics of the reflectance spectra of pear leaves are shown in Figure 2. The distribution of the spectra obtained using different methods followed roughly the same trend. However, the spectra measured by the 25° field of view method showed a low signal-to-noise ratio, especially in the two water band noise regions of 1440 nm and 1900 nm. Moreover, the reflectance at 2000–2500 nm had abnormally low reflectivity compared to the other two methods (Figure 2a). Spectra measured by using a leaf clip with black and white backgrounds represented absolute reflectance and transflectance, respectively. Due to the additional transmittance, reflectance measured with the white background was generally higher than that measured by the black one, especially in the regions of 800 to 1300 nm, 1550 to 1850 nm, and 2200 nm (Figure 2b).

**Figure 3:**
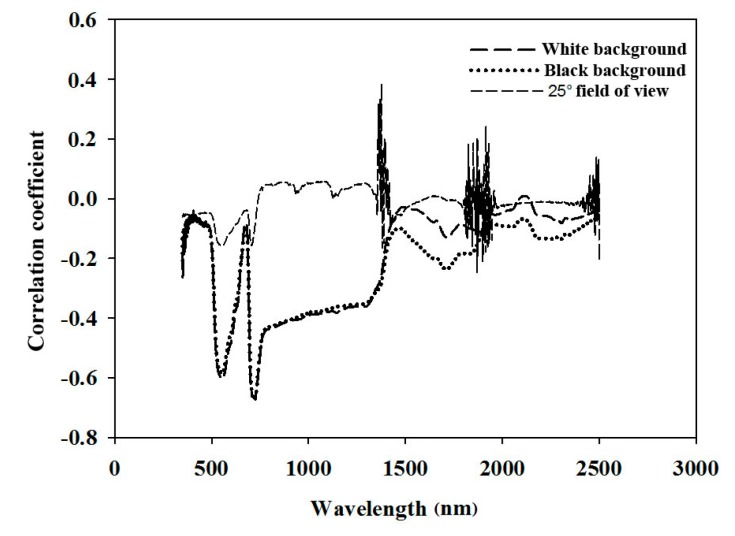
Correlation coefficients between the chemically measured N concentration in pear leaves and reflectance measured by the three methods.

The correlations between the reflectance measured by the three methods and N concentration are shown in Figure 3. 

Except for the low correlation of reflectance obtained by the 25° field of view method, the remaining two methods were relatively highly correlated with the leaf N concentration. In addition, the N concentration was highly correlated with the reflectance regions of 500–640 nm and 690–1380 nm. In the visible and near-infrared band (350–1100 nm), the correlation between the reflectance and N concentration of the methods with white and black backgrounds was similar, whereas the spectral reflectance measured by the black background better reflected the correlation with the leaf N concentration than that measured by the white background in the short-wave infrared band (1500–2500 nm). 

### 3.2. Performances of Four Chemometrics 

Due to the low correlation of reflectance obtained by the 25° field of view method (Figure 3), all of the modelling was performed on two spectral datasets—both acquired by a plant probe. From Table 2, the reflectance spectra with the black background were better at both modelling and predicting than the transflectance measured with the white background (R^2^ was higher, and RMSE was lower). Comparing the results of the four chemometric methods, the modelling accuracy of PCA was significantly lower (R^2^ = 0.40 to 0.42) than that of SMLR, PLSR, and BPNN (R^2^ = 0.76 to 0.89). In contrast, the prediction accuracy of BPNN (R^2^ = 0.67) decreased significantly compared to its modelling accuracy (R^2^ = 0.89). The modelling accuracy of BPNN could be improved by increasing the principal components and the number of iterations. However, the prediction accuracy did not increase with the increasing of principal components and number of iteration in the validation process. In this situation, the BPNN modelling method easily over-fitted the dataset. SMLR and PLSR showed good modelling and prediction accuracy. In conclusion, PLSR, combined with the spectral reflectance as measured with the black background, produced the best modelling accuracy. The optimal number of factors by leave-one-out cross validation is fifteen.

### 3.3. Modelling the Three Vegetation Indices

**Figure 4:**
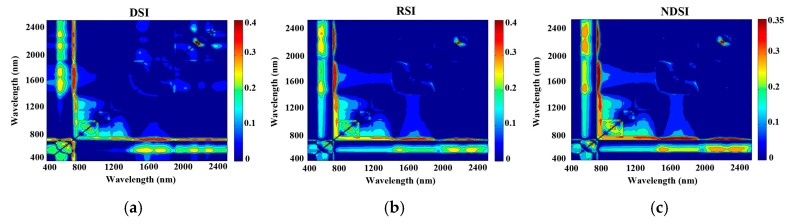
Contour maps of coefficients of determination for the linear relationship between the three vegetation indices (difference spectral index (DSI, **a**), ratio spectral index (RSI, **b**), or normalized difference spectral index (NDSI, **c**)) and chemically-measured leaf N concentration using the spectra measured by the black background.

**Figure 5:**
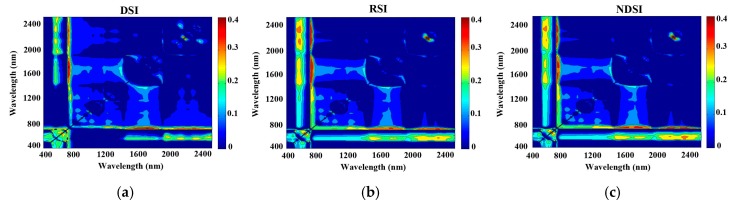
Contour maps of coefficients of determination for the linear relationship between three vegetation indices (DSI (**a**); RSI (**b**); or NDSI (**c**)) and the chemically-measured leaf N concentration using the spectra measured by the white background.

The reduced sampling method was used to analyse the relationships between N concentration and DSI, RSI, and NDSI at 10 nm intervals from 350 nm to 2500 nm based on the pre-processed spectrum to identify the sensitive bands with high R^2^ values. The contour map of R^2^ (Figure 4 and Figure 5) revealed significant correlations between the N concentration in pear leaves and the three indices (R^2^ ranged from 0.39 to 0.44 with the black or white background). As seen from the wavelength of the max R^2^ with spectra measured by the white background, the R^2^ values were greater than those with the black background, according to the linear regression based on RSI and NDSI of 2150 nm and 2170 nm, respectively (Table 3). In contrast, the wavelength of the max R^2^ with spectra measured by the black background was greater than that with the white background, according to the linear regression based on DSI of 2170 nm and 2150 nm. The DSI of the black background had the highest R^2^ among the three indices.

### 3.4. Two-Year Nitrogen Status as Dependent on the N Treatment

**Figure 6:**
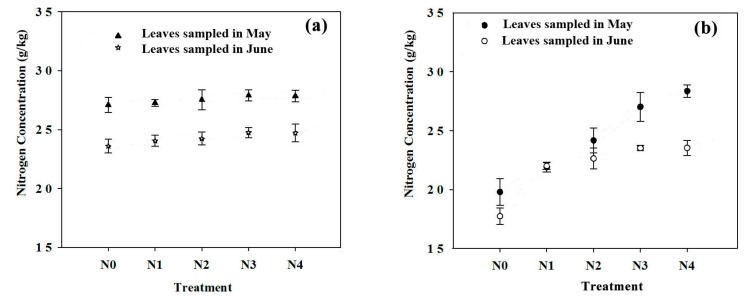
Leaf nitrogen concentrations at the fruit expansion stage in 2014 (**a**) and 2015 (**b**). Every point is an average of 60–80 leaves. Vertical bars represent ± SE.

The leaf N concentration varied from 23–27 g/kg in 2014 (Figure 6a) and from 17–28 g/kg in 2015 (Figure 6b) depending on the fertilizer treatment. In 2015, increased leaf N concentrations in both May and June were found with the increased N application rate. From May to June, the leaf N concentration in both 2014 and 2015 decreased by approximately 5 g/kg on average due to a progression from the vegetative to reproductive stages. 

### 3.5. Single-Fruit Weight and Yield per Tree as Dependent on the N Treatment

**Figure 7:**
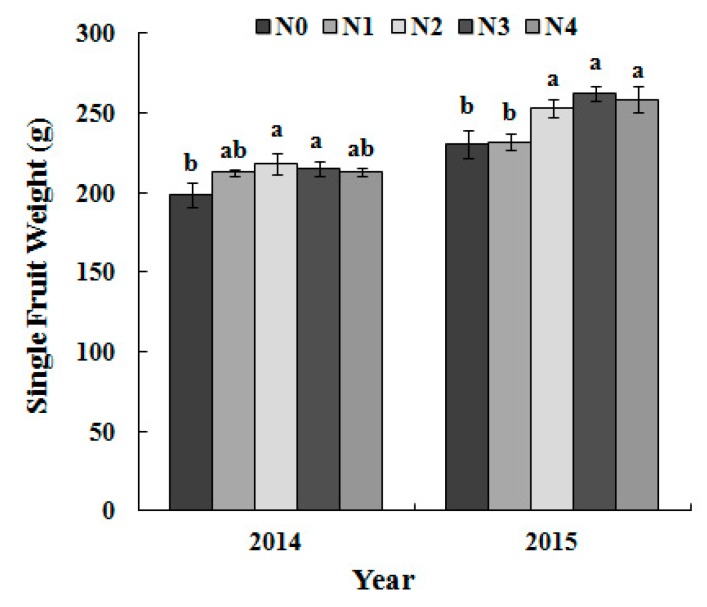
Single fruit weights in different N treatments.

**Figure 8:**
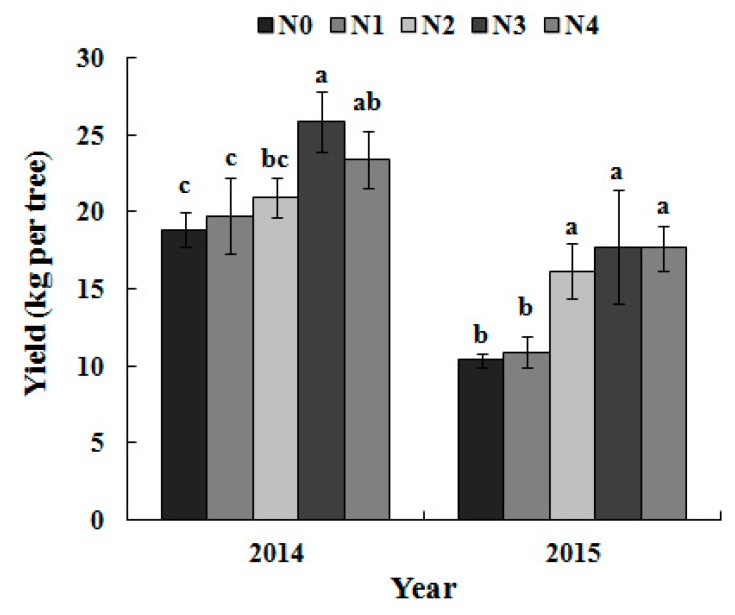
Fruit yields in different N treatments.

Single-fruit weight and the yield increased at first and then decreased with the N application rate in 2014. However, no significant increase was found in yield and single-fruit weight with an increase in the N application rate beyond N2 in 2015 (Figure 7 and Figure 8). 

The fruit size of a pear is one of its most important commercial attributes; thus, the single fruit weight (200–250 g) was controlled by thinning. The yield in 2015 was relatively low because of biennial fruiting.

### 3.6. Performance of the Best PLSR Model Tested with Samples Collected in Two Years

**Figure 9:**
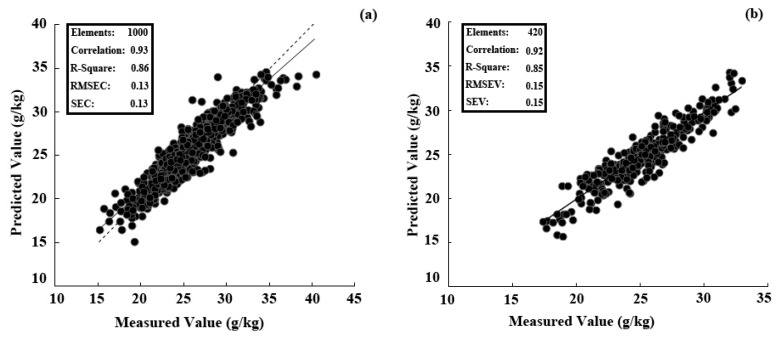
Measured vs. predicted N concentration for calibration (**a**) and validation (**b**) by the best partial least squares (PLS) regression model (factor = 15) with spectra measured using the black background. The solid line of (**a**) represented the best regression line and the dashed line of (**a**) represented the target line (1:1 line). The best regression line (solid line) and the target line (dashed line) in the validation set almost showed superposition.

Compared with the seven kinds of modelling methods (four chemometric methods and three vegetation indices) above, combined with two different spectra measurements (Table 2), the spectra measured with the black background combined with the PLSR showed the best modelling and prediction accuracy. The sample set from two years was randomly divided into the calibration set (*n* = 1000) and the validation set (*n* = 420); the model developed here was based on the PLSR with 15 factors (Figure 9). As shown in Figure 9a, the solid line represents the best regression line and the dashed line represented the target line (1:1 line). However, the best regression line and the target line in the validation set almost showed superposition. The coefficient of determination (R^2^) of the chemically-measured N concentration in leaves and predicted values was >0.85 in both the calibration and validation sets, indicating that the model can be used to predict the N concentration in pear leaves.

### 3.7. Relationship Between the Leaf Nitrogen Concentration and Yield

**Figure 10:**
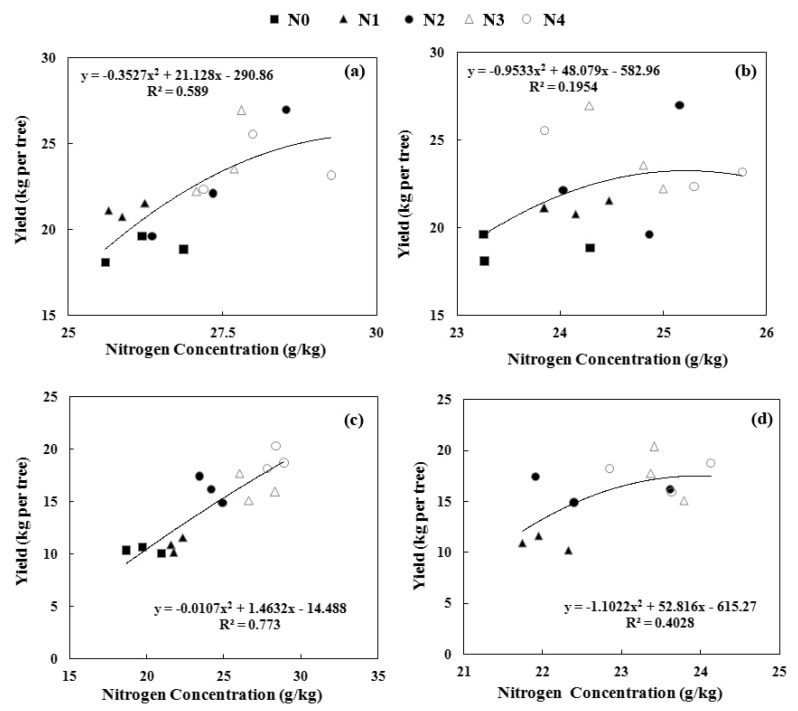
Relationship between the chemically-measured leaf N concentration and the fruit yield. Leaf samples collected in May (**a**) and June (**b**) 2014 as well as May (**c**) and June (**d**) 2015. Each point is an average of three trees.

The chemically-measured leaf N concentration was better related to the yield in 2015 than 2014 (Figure 10), probably because of remobilization of N in the early spring of 2014. However, compared to the two sampling times, the earlier sampling (May) was better in both 2014 (R^2^ = 0.59) and 2015 (R^2^ = 0.77) than the later sampling (June), suggesting that an early diagnosis was more accurate and may ensure timely fertilizer application.

## 4. Discussion 

### 4.1. In-Field Spectral Measurement of Pear Leaves

It is necessary to establish a non-destructive, real-time leaf N determination method to predict the N status to better manage N nutrition and gain higher yields and fruit quality in pear orchards. The results of this work showed that leaf N status can be well predicted in the field, using a leaf-clip with a black background attached to a plant probe based on single-leaf spectral reflectance. Compared to the other two spectral measurements tested in this study, the leaf N concentration was more closely related to the leaf spectral reflectance measured by the black background based on the correlation coefficient and modelling accuracy. This result might be related to the lack of leaf dust removal in the real-time spectral measurement of pear leaves. The underside of the leaf retains dust more than the leaf surface, which might have resulted in relatively low signal-to-noise spectra when measured with the white background, especially in the short-wave infrared region. Light reflected by vegetation in the VIS region of the spectrum (350–750 nm) in remote sensing experiments is predominantly influenced by chlorophyll pigments in the leaf tissues [39]. However, the short-wave infrared region was highly correlated with material absorption especially some structural leaf compounds, such as cell walls, cellulose, and lignin [40,41], leaf cellular structure, such as the volume of the air spaces, and the direction of incoming light [42]. Spectral measurement using the black background can minimize the influence of dust on the underside of leaves, and was found to be more related to the leaf nitrogen concentration. 

### 4.2. Comparison of Modelling Methods 

It is inevitable that spectra are collected that contain not only information related to leaf N concentration, but also information in response to the sample state (especially the water deficit and physiological activities) because this is an in-field measurement of fresh leaves on a tree growing in a complex environment. It is more difficult to extract characteristic wavelengths in the field compared to the steady conditions in the lab as done in previous studies [11,43]. Due to the low correlation of reflectance obtained by the 25° field of view method, all of the modelling was performed on two spectral datasets—both acquired by a plant probe. Our results showed that the “full-spectra” method of PLSR was better than the SMLR and vegetation indices, which were consistent with Nguyen and Lee [44] that the PLSR can alleviate the problem of high dimensionality by seeking a parsimonious number of factors through a linear projection of original bands. Although the R^2^ is relatively low and the wavelengths were probably highly correlated, Figure 4 and Figure 5 showed the importance of the red-edge region for N estimating. Nevertheless, vegetation indices, such as the RVI and NDVI, are still widely used mainly because of their simplicity and high correlation to the pigment [45,46,47]. 

Among the three “full-band” modelling methods, PLSR (R^2^ = 0.81) was superior to PCR (R^2^ = 0.41) and BPNN (R^2^ = 0.67) in prediction accuracy. PCR accounts for only the variance of explanatory variables (e.g., band reflectance) without considering any response variable (e.g., biochemical concentration), whereas PLSR accounts for both [17,18]. BPNN performed well in modelling the soluble solid content of navel orange fruit in the spectral measurements in the lab [35]. However, in the present study with the complex in-field measurements, the first step by PCA was not that useful for fitting the leaf N concentration to the variable input in the BPNN. Further research should insist on conquering the limitations using a support vector machine or genetic algorithm with the BPNN [48]. Considering the calibration and validation, the PLSR provided the most accurate performance in the real-time spectral measurements to determine leaf N concentration in pear trees among the seven modelling methods with the best accuracies (R^2^ > 0.85 and mean relative error <6%). The best regression line in the calibration set showed a deviation from the target line, which may be the correction of systematic error in the modelling procedure. The best regression line and the target line in the validation set almost showed superposition, which illustrated the good predictive ability of this model.

### 4.3. Threshold of Leaf N Concentration and Right Diagnostic Time

Decisions about when to apply N and the proper N application rate were needed for the practical management of pear orchards. With fruit development, the leaf N concentration changed in both years. Especially in 2015, leaf N concentration was found to increase with the increased N application rate. As woody plants, pear trees use two main sources of N to sustain vegetative and reproductive growth: N remobilization from storage and root N uptake [5]. In 2014, at the time of leaf collection, N remobilization of the last year was the predominant source of spur leaves and flowers [5,49], which leads to an N concentration almost independent from the treatment. Furthermore, the N concentration was higher in May than in June sampling due to progression from the vegetative to reproductive stages. This period is a key period in fruit growth, and non-destructive diagnosis of the N status is important for timely fertilizer application for fruit quality and yield. Previous studies [5,34] have suggested that, after bloom, the earlier the leaf N level was diagnosed the better so that an increasing number of methods can be adopted to increase or decrease the leaf N concentration as soon as possible. In the present study, a closer relationship was found between the N concentration and fruit yield in the May sampling (both in 2014 and 2015) than in the June sampling (Figure 10). 

Fruit yield did not significantly increase with the increased N application rate beyond the N2 rate (Figure 7 and Figure 8). This “excess applied N” may be used in the extra vegetative growth of trees, such as trunk enlargement or increased elongation of shoots that easily grow on pear trees managed by trellis cultivation, leading to additional work during the annual winter pruning. Considering the management practices regarding tree nutrient loss (especially in annual winter pruning), the optimum N application rate should range from N2 (330 kg·N·ha^−1^) to N3 (660 kg·N·ha^−1^), which was a decrease by 50% compared to the local N application ratio. The best diagnosis time of the N status should be the early fruit expansion stage (May), and the reference threshold of the leaf N concentrations should be 24–27 g/kg in the tested orchard. 

Compared to other research on the leaf N determination by spectral measurement [9,50] or the relatively low accuracy of yield estimation by hyperspectral measurements [29,30], our method provides an easy and flexible way to not only guide N fertilization, but also estimate the current yield. However, this experiment was performed with variable N applications, potash and phosphate fertilizer, not allowing an assessment of tree responses to a variety of nutrient deficiencies or excesses. To allow the large-scale application of the method, it is essential (i) to establish the reliability of this method under a variety of conditions, such as water and/or nutrient deficits, growth regulator application, different cultivars, etc.; and (ii) to establish a robust leaf N concentration threshold, below which yield is expected to decrease. Once these requirements are met, the method could be safely used for on-the-spot decision-making regarding the N fertilization of pear trees to prevent excessive N application for an increased benefit/cost ratio and the protection of the environment. Further work should also include testing of other methods (such as support vector machines and genetic algorithms) to extract the characteristic wavelengths while reducing the redundancy in computation.

## 5. Conclusions

In this study, the utilization of in-field visible/near-infrared spectral measurements realized the non-destructive nitrogen determination of pear leaves. Spectra collected by the plant probe with the black background were found to provide the highest signal-to-noise ratio. The model established by the partial least squares regression resulted in high modelling and prediction accuracy. A higher coefficient of determination between the leaf N concentration and fruit yield was found at 50 days after full bloom.

## Figures and Tables

**Table 1:** Chemically measured nitrogen concentration in pear leaves (2014) used for modelling calibration and validation.

Dataset	Samples Collected in May				Samples Collected in June			
	Sample No.	Min.	Max.	Mean	Sample No.	Min.	Max.	Mean
		(g/kg)				(g/kg)		
**Total**	340	18.7	35.2	25.4 ± 3.0	370	20.3	30.9	24.7 ± 1.9
**Calibration**	290	20.5	35.2	27.4 ± 2.7	310	20.3	30.9	24.8 ± 2.0
**Validation**	50	18.7	30.2	23.4 ± 2.4	60	21.4	28.2	24.5 ± 1.7

**Table 2:** Modelling results using four chemometric methods with two spectral datasets as measured by black or white backgrounds.

Method ^†^	Black Background				White Background			
	Calibration		Validation		Calibration		Validation	
	R^2^	RMSEC	R^2^	RMSEV	R^2^	RMSEC	R^2^	RMSEV
PCR	0.42	0.23	0.41	0.24	0.42	0.25	0.40	0.26
SMLR	0.78	0.14	0.75	0.19	0.77	0.14	0.73	0.20
PLSR	0.86	0.12	0.81	0.13	0.82	0.13	0.78	0.15
BPNN	0.89	0.23	0.67	0.17	0.88	0.24	0.61	0.18

^†^ PCR, SMLR, PLSR and BPNN represent principal component regression, stepwise multiple linear regression, partial least-squares regression and back propagation neural networks, respectively.

**Table 3:** Linear regression between three vegetation indices and chemically measured leaf nitrogen concentration.

Vegetation Index ^††^	Black Background		White Background	
	R^2^	Wavelength of Max R^2^	R^2^	Wavelength of Max R^2^
DSI	0.46	2170 nm, 2150 nm	0.41	1690 nm, 710 nm
RSI	0.40	1710 nm, 720 nm	0.44	2170 nm, 2150 nm
NDSI	0.39	1690 nm, 730 nm	0.44	2150 nm, 2170 nm

^††^ DSI, RSI and NDSI represent difference spectral index, ratio spectral index and normalized differential spectral index, respectively.
